# Differences in total and differential white blood cell counts and in inflammatory parameters between psychiatric inpatients with and without recent consumption of cannabinoids, opioids, or cocaine: A retrospective single-center study

**DOI:** 10.1016/j.bbih.2024.100898

**Published:** 2024-11-06

**Authors:** Vicent Llorca-Bofí, Maria Mur, Maria Font, Roberto Palacios-Garrán, Maite Sellart, Enrique del Agua-Martínez, Miquel Bioque, Gara Arteaga-Henríquez

**Affiliations:** a Department of Psychiatry, Hospital Universitari Santa Maria, Lleida, Spain; b Department of Medicine, University of Barcelona, Barcelona Clínic Schizophrenia Unit (BCSU), Neuroscience Institute, Hospital Clínic de Barcelona, Barcelona, Spain; c Department of Medicine and Surgery, Universitat de Lleida, Institut de Recerca Biomèdica de Lleida (IRBLleida), Spain; d Laboratory Department, Arnau de Vilanova University Hospital, Lleida, Spain; e Mental Health Unit, Hospital Universitario Jerez de la Frontera, University of Cádiz, Cádiz, Spain; f Barcelona Clínic Schizophrenia Unit (BCSU), Neuroscience Institute, Hospital Clínic de Barcelona, Institut d'Investigacions Biomèdiques August Pi i Sunyer (IDIBAPS), Department of Medicine, University of Barcelona, Barcelona, Spain; g Biomedical Network Research Centre on Mental Health (CIBERSAM), Madrid, Spain; h Department of Psychiatry, Hospital Universitari Vall d'Hebron, Vall d'Hebron Research Institute (VHIR), Vall d'Hebron Barcelona Hospital Campus, Barcelona, Spain; i NCRR-National Center for Register-based Research, Aahrus University, Aahrus, Denmark

**Keywords:** Schizophrenia, Inflammation, Cannabinoids, Cocaine, Opioids, Drugs of abuse, Neutrophils

## Abstract

Several drugs of abuse may exert their action by modulating the immune system. Despite this, individuals using substances of abuse are often excluded from immunopsychiatry studies. We conducted a retrospective, single-center study to examine differences in circulating immune/inflammatory parameters (i.e., total and differential white blood cell (WBC) counts, neutrophil-to-lymphocyte ratio, monocyte-to-lymphocyte (MLR) ratio, platelet-to-lymphocyte ratio, and C-reactive protein) between psychiatric inpatients with a positive urine test to cannabinoids, opioids, or cocaine, and those with negative toxicology. A total of 927 inpatients were included. Patients with positive toxicology (n = 208) had significantly higher WBC counts (*P* < 0.001, *η*^2^p = 0.02), as well as increased neutrophils (*P* = 0.002, *η*^2^p = 0.01), monocytes (*P* < 0.001, *η*^2^p = 0.02), lymphocytes (*P* < 0.001, *η*^2^p = 0.02), and eosinophils (*P* = 0.01, *η*^2^p = 0.01) compared to those with negative toxicology (n = 719). The increase in neutrophil counts was particularly evident in patients who tested positive for cannabinoids (n = 168; *P* < 0.001, *η*^2^p = 0.02). In contrast, eosinophil counts were particularly increased in the cocaine-positive subgroup (n = 27; *P* = 0.004, *η*^2^p = 0.01). Patients with a positive urine test to opioids (n = 13) were characterized by a significantly lower MLR (*P* = 0.03, *η*^2^p = 0.005). The type of psychiatric diagnosis moderated the differences in neutrophil counts between patients with a positive and negative toxicology to cannabinoids. Notably, significantly higher neutrophil counts were found only in patients diagnosed with a psychotic disorder (*P* < 0.001, *η*^2^p = 0.03). Taken together, our findings suggest that drugs of abuse may differently impact the immune/inflammatory response system in individuals diagnosed with psychiatric conditions. Specifically, recent cannabinoids use may be associated with an acute activation of the inflammatory response system, particularly in individuals with a psychotic disorder, while cocaine and opioid use may be associated with eosinophilia and a decrease in the MLR, respectively, regardless of the primary psychiatric diagnosis.

## Highlights

• Cannabinoids, opioids, and cocaine use may differently impact the immune system in psychiatric inpatients.
• Patients under cocaine use showed higher eosinophil counts.
• Patients under opioids use showed a decreased MLR.
• Higher neutrophil counts were particularly observed in patients with psychotic disorders using cannabinoids.

## 1 Introduction

Accumulating evidence suggests that immunological and/or inflammatory changes may underly several psychiatric conditions, such as schizophrenia, major depressive disorder, bipolar disorder, autism spectrum disorder and/or personality disorders. An increased expression of different pro-inflammatory genes, abnormalities in the number and/or function of several immune cells (e.g., monocytes, lymphocytes), and increased blood and/or cerebrospinal fluid levels of pro-inflammatory compounds (e.g., cytokines, chemokine and/or C-reactive protein (CRP)) have been repeatedly reported in individuals diagnosed with these conditions (Goldsmith et al., 2016; Vogels et al., 2017; Becking et al., 2018; Garcia-Rizo et al., 2019; Jackson and Miller, 2020; Simon et al., 2023; Bioque et al., 2022; Arteaga-Henríquez et al., 2022; López-Villatoro et al., 2023; Sørensen et al., 2023). The relevance of these findings lies in their potential use as biological diagnostic and therapeutic markers in individuals with psychiatric symptoms (personalized psychiatry) (Drexhage et al., 2010; Benedetti et al., 2017; Becking et al., 2018; Arteaga-Henríquez et al., 2019; Ioannou et al., 2024; Llorca-Bofí et al., 2024). For a better understanding of the whole picture, it is important to investigate how several factors, such as age (Grosse et al., 2015), previous viral infections (Simon et al., 2023), childhood trauma (Schiweck et al., 2020) and/or obesity (Milaneschi et al., 2020; Arteaga-Henriquez et al., 2021) may influence the immune/inflammatory changes found in individuals diagnosed with psychiatric conditions (Pillinger et al., 2019).

Substance abuse is highly comorbid in individuals diagnosed with psychiatric disorders (Ross and Peselow, 2012; Toftdahl et al., 2016), i.e., about 10–50% of the subjects diagnosed with a psychiatric disorder will also experience a comorbid substance use disorder (SUD) at some point in their lives (Ross and Peselow, 2012; Toftdahl et al., 2016). Accumulating research suggests that individuals who use substances of abuse may be at a higher risk of treatment resistance and suicidal behavior. On the contrary, other reports also suggest the potential beneficial effect of substances like cannabinoids in the management of anxiety (Schott, 2019). The mechanisms by which these agents impact mood and behavior are not fully understood but may be related to their capability to impact the immune/inflammatory response system exerting, depending on the agent and probably, on the target population, and anti-inflammatory and/or pro-inflammatory action (Tanasescu and Constantinescu, 2010; Bidwell et al., 2020; Henshaw et al., 2021). Despite this, patients meeting criteria for a SUD or under sporadic substance of abuse use are frequently excluded from Immunopsychiatry studies representing an important gap in the literature.

The aim of this retrospective study was to investigate the differences in relation to the levels of a set of immune/inflammatory parameters (i.e., total and differential white blood cell (WBC) counts, platelet counts, neutrophil-to-lymphocyte ratio (NLR), lymphocyte-to-monocyte ratio (LMR), platelet-to-lymphocyte ratio (PLR), and CRP), between patients with a psychiatric diagnosis and a positive toxicology to cannabinoids, opioids or cocaine and those with a negative toxicology.

## 2 Material and methods

### 2.1 Study participants

For this retrospective study, electronic medical records of all patients admitted to the Inpatient Psychiatric Unit (Santa María University Hospital, Lleida, Spain) between January 1, 2010, and December 31, 2020, were extensively reviewed by two experienced psychiatrists. In order to avoid duplications (and also, to indirectly control for other factors, such as chronicity and/or polypharmacy), we only included data referred to the first time of admission.

Included were acutely ill psychiatric inpatients aged 18 or over, from whom a blood and urine sample was collected at admission (i.e., within the first 24 h). Excluded were subjects diagnosed with a psychiatric disorder secondary to a known medical condition or with a non-specified psychiatric disorder. Patients admitted after a suicide attempt, pregnant or breastfeeding women, as well as individuals diagnosed with a comorbid autoimmune and/or acute/chronic inflammatory, metabolic, cardiovascular, or neurological (including neurocognitive) condition were excluded, too. Subjects testing positive for other agents of abuse than benzodiazepines (BZD), cannabinoids, opioids and/or cocaine (i.e., those testing positive for amphetamines/amphetamine derivatives) were also not included. Given the described association between alcohol consumption and immune system/inflammatory dysfunction (Calleja-Conde et al., 2021), subjects under an alcohol substance use disorder and/or under alcohol use were excluded.

The study protocol was approved by the Local Ethics Committee belonging to the Santa María's University Hospital (Lleida, Spain).

### 2.2 Study procedures

Blood and urine were collected between 8.00 and 10.00 a.m. by an experienced nurse belonging to the Department of Psychiatry at Santa María's University Hospital (Lleida, Spain) after an overnight fasting. Selected blood markers included: total and differential WBC counts (i.e., basophils, eosinophils, neutrophils, monocytes, lymphocytes), platelet counts and CRP levels. In addition, the following indexes were calculated: NLR, MLR, and PLR. Total and differential WBC counts were assessed by flow cytometry using a Sysmex XN analyzer; the detection range, as determined by the assay manufacturer was set at 0-440 × 10^9^/L. Platelet counts were assessed by impedance also by a Sysmex XN analyzer; the detection range was in this case set at 0-5000 × 10^9^/L. CRP levels were assessed by an immunoturbidimetric assay on a Beckman Coulter automated analyzer; the lower limit of detection, as determined by the assay manufacturer was set on 2 mg/L. Positivity in urine (no/yes) was determined by immunochromatography (i.e., Multi-line Drug Screen Test Device (MONLAB)), and established according to both Substance Abuse Mental Health Administration (SAMHSA) (Verstraete and Pierce, 2001), and United Nations International Drug Control Program criteria (SAMHSA, 2004). The cut-off limits for considering a patient as “positive” were 50 ng/mL for cannabinoids, 300 ng/mL for opioids and 300 ng/mL for cocaine (Verstraete and Pierce, 2001).

### 2.3 Statistical analyses

Statistical analyses were performed using IBM-SPSS v.23 (IBM SPSS Statistics for Windows, Armonk, NY: IBM Corp., USA). Continuous data were expressed as mean ± standard deviation (SD), while categorical data were expressed as absolute values and percentages (%). Data were tested for normal distribution by the Kolmogorov-Smirnov test (n ≥ 30). Group comparisons of sample characteristics were analyzed by using Mann-Whitney *U* tests (i.e., continuous data), or Pearson's chi-square (*χ*^2^) tests (i.e., categorical data). A univariate analysis of covariance (ANCOVA) model was used to compare mean immune/inflammatory parameter levels between patients with a negative vs. those with a positive toxicology. Age (years), consumption of BZD (no/yes) and the type of primary psychiatric diagnosis were introduced as covariables. Sensitivity subgroup analyses were additionally performed, in order to explore the effect of the type of agent of use, as well as the type of primary psychiatric diagnosis on immune/inflammatory parameters. Effect sizes are reported as partial eta-squared (*η*^*2*^p). All tests were two-tailed, with *P*-values equal or less than 0.05 being considered as of statistical significance.

## 3 Results

### 3.1 Baseline characteristics of study participants

Baseline characteristics of the 927 patients included are reported in Table 1. For a complete description of the diagnoses included, see Table S1.

**Table 1:** Sample characteristics of study participants.

	Negative n = 719	Positive n = 208	Negative vs. Positive:	Cannabinoids n = 168	Negative vs. Positive:	Opioids n = 13	Negative vs. Positive:	Cocaine n = 27	Negative vs. Positive:
Age (years); mean (SD)	44.93 (16.16)	35.11 (10.75)	**<0.001**	33.89 (10.25)	**<0.001**	42.95 (15.12)	0.66	38.93 (9.22)	0.06
Females; n (%)	366 (51%)	111 (53%)	0.53	91 (54%)	0.45	9 (69%)	0.19	11 (41%)	0.30
Agent of use, n (%)									
- BZD	396 (55%)	136 (66%)	**0.01**	105 (63%)	0.08	9 (69%)	0.32	22 (81%)	**0.005**
- Cannabinoids	0 (0%)	168 (81%)							
- Opioids	0 (0%)	13 (6%)							
- Cocaine	0 (0%)	27 (13%)							
Primary Psychiatric; n (%)									
- SUD	0 (0%)	46 (22%)	**<0.001**	29 (17%)	**<0.001**	5 (38%)	**<0.001**	12 (44%)	**<0.001**
- Psychotic Disorder	312 (43%)	81 (39%)	0.25	73 (43%)	0.99	1 (8%)	**0.01**	7 (26%)	0.07
- Depressive Disorder	108 (15%)	7 (3%)	**<0.001**	4 (2%)	**<0.001**	3 (23%)	0.31	0 (0%)	**0.01**
- Bipolar Disorder	130 (18%)	26 (12%)	0.06	23 (14%)	0.17	2 (15%)	0.57	1 (4%)	**0.03**
- Adjustment Disorder	85 (12%)	14 (7%)	**0.02**	10 (6%)	**0.03**	2 (15%)	0.47	2 (7%)	0.48
- PD	46 (6%)	23 (11%)	**0.02**	18 (11%)	**0.05**	0 (0%)	0.43	5 (18%)	**0.03**
- NDV	7 (1%)	1 (1%)	0.43	1 (1%)	0.64	0 (0%)	0.88	0 (0%)	0.77
- OCD	7 (1%)	0 (0%)	0.17	0 (0%)	0.23	0 (0%)	0.88	0 (0%)	0.77
- Eating Disorder	17 (2%)	1 (1%)	0.06	1 (1%)	0.12	0 (0%)	0.73	0 (0%)	0.53
- Conduct Disorder	7 (1%)	9 (4%)	**0.001**	9 (5%)	**0.001**	0 (0%)	0.88	0 (0%)	0.77

*Abbreviations:* BZD: benzodiazepines; NDV: Neurodevelopmental Disorder; OCD: Obsessive-Compulsive Disorder; PD: Personality Disorder; SD: standard deviation; SUD: Substance Use Disorder. *P*-values were based on Mann Whitney *U* tests (continuous variables), and on χ^2−^Squared test (categorical variables). Significant values are highlighted in bold.

In total, 719 (78%) of patients had a negative toxicology; 208 (22%) had a positive toxicology (i.e., cannabinoids (n = 168, 18%), opioids (n = 13, 1%), cocaine (n = 27, 3%)). Among the total of patients with a positive toxicology, 46 (22%) were diagnosed with a SUD.

Patients with a positive toxicology were statistically significantly younger (*P<*0.001) and showed a statistically significantly higher consumption of BZD (*P* = 0.01) compared to those with a negative urine test. In addition, subjects with a positive toxicology were diagnosed with a conduct (i.e., 9% vs 4%; *P* = 0.001) or a personality disorder (i.e., 11% vs. 6%; *P* = 0.02) at a statistically significantly higher proportion; and with a depressive (i.e., 3% vs. 15%; *P* < 0.001) or an adjustment disorder (7% vs. 12%; *P* = 0.02) at a statistically significantly lower proportion, (Table 1).

Significant differences were not found between patients testing negative and those testing positive in relation to sex or to the proportion of psychotic, bipolar, neurodevelopmental, obsessive-compulsive or eating disorder diagnoses (Table 1).

### 3.2 Blood levels of immune/inflammatory parameters in individuals with a negative urine test vs. individuals with a positive urine test

Overall, patients with a positive toxicology were characterized by a statistically significantly higher WBC count (*P* < 0.001, *η*^2^p = 0.02) and by statistically significantly higher blood levels of neutrophils (*P* = 0.002, *η*^2^p = 0.01), monocytes (*P* < 0.001, *η*^2^p = 0.02), lymphocytes (*P* < 0.001, *η*^2^p = 0.02) and eosinophils (*P* = 0.01, *η*^2^p = 0.01) compared to those with a negative toxicology (Table 2). Patients with a positive toxicology were on the contrary characterized by a statistically significantly lower PLR (*P* = 0.01, *η*^2^p = 0.01) compared to those testing negative (Table 2). Significant differences were not found between patients with a positive toxicology and those with a negative toxicology in relation to any of the other immune/inflammatory parameters assessed (Table 2).

**Table 2:**
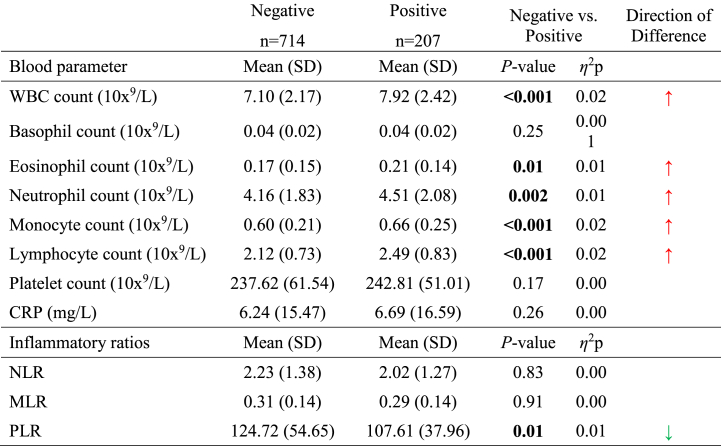
Blood levels of immune/inflammatory parameters in individuals with a negative urine test vs. individuals with a positive urine test.

*Abbreviations*: CRP: C-reactive protein; MLR: monocyte-to-lymphocyte ratio; NLR: neutrophil-to-lymphocyte ratio; PLR: platelet-to-lymphocyte ratio; WBC: white blood cell count. Analyses were based on an analysis of covariance (ANCOVA) model with the corresponding blood parameter as the dependent variable, group (negative/positive) as fixed effect variable, and age, consumption of BZD (yes/no), and the type of primary psychiatric diagnosis, as covariates. Significant *P*-values are highlighted in bold and marked with an asterisk (i.e., ∗∗∗P≤ 0.001, ∗∗P≤ 0.01, ∗P≤ 0.05).

### 3.3 Blood levels of immune/inflammatory parameters in individuals testing negative vs. those testing positive, stratified by the agent of use

**Fig. 1:**
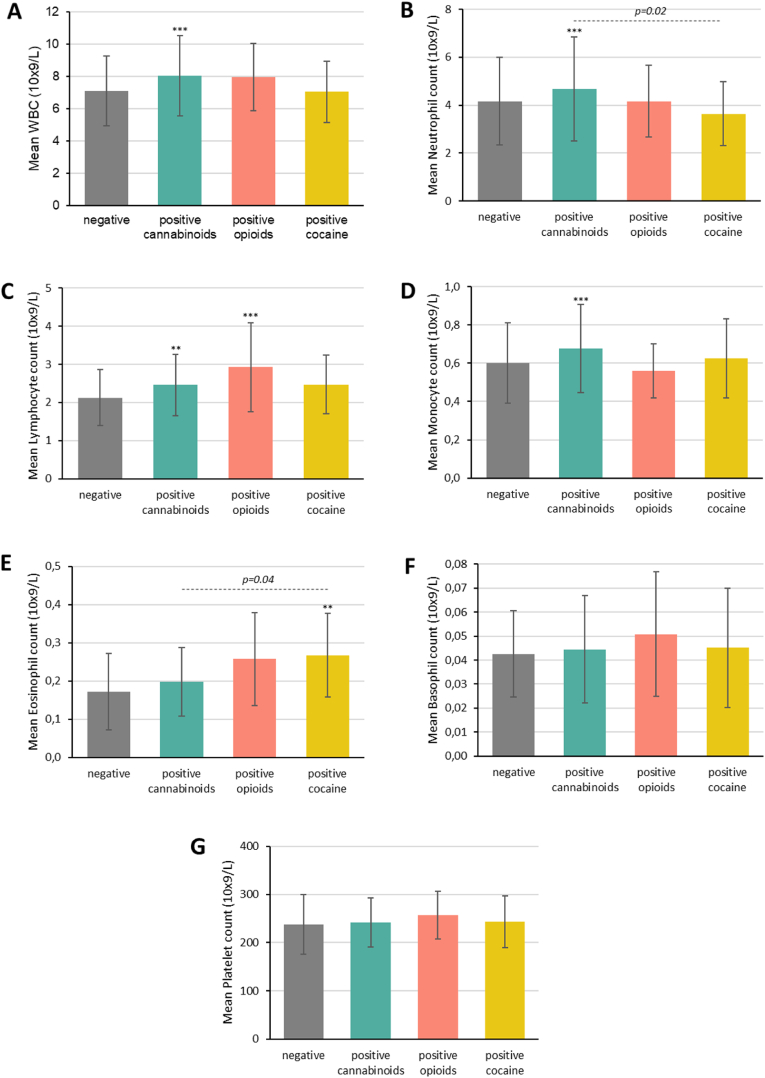
Blood levels of immune parameters in individuals with a negative urine test vs. individuals with positive urine test (stratified by agent of consumption; cannabinoids, opioids, or cocaine). *Abbreviations*: WBC: white blood cell count. Analyses were based on an analysis of covariance (ANCOVA) model with the corresponding blood parameter as the dependent variable, group as fixed effect variable, and age, consumption of BZD, and type of primary psychiatric diagnosis, as covariates. Significant values are marked with an asterisk (i.e., ∗∗∗P ≤ 0.001, ∗∗P ≤ 0.01, ∗P ≤ 0.05) when comparing negative vs. specific positive urine tests, and with the corresponding p-value (---) when comparing the positive urine tests among themselves.

**Fig. 2:**
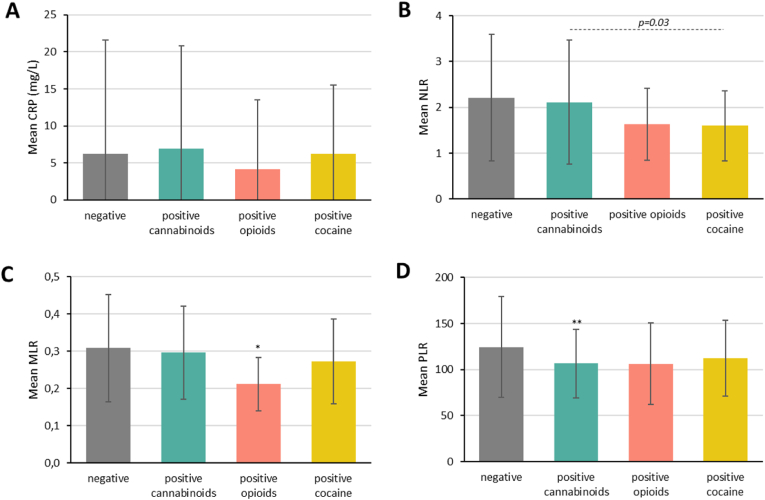
Blood levels of inflammatory parameters in individuals with a negative urine test vs. individuals with positive urine test (stratified by agent of consumption; cannabinoids, opioids, or cocaine). *Abbreviations*: CRP: C-reactive protein; MLR: monocyte-to-lymphocyte ratio; NLR: neutrophil-to-lymphocyte ratio; PLR: platelet-to-lymphocyte ratio. Analyses were based on an analysis of covariance (ANCOVA) model with the corresponding blood parameter as the dependent variable, group as fixed effect variable, and age, consumption of BZD, and type of primary psychiatric diagnosis, as covariates. Significant values are marked with an asterisk (i.e., ∗∗∗P ≤ 0.001, ∗∗P ≤ 0.01, ∗P ≤ 0.05) when comparing negative vs. specific positive urine tests, and with the corresponding p-value (---) when comparing the positive urine tests among themselves.

As a sensitivity analysis, we stratified patients with a positive urine test according to their agent of use and then compared them to those with a negative urine test (Fig. 1, Fig. 2, Table S2).

By doing so, we found that patients with a positive toxicology to cannabinoids were characterized by a statistically significantly higher WBC count (*P* < 0.001, *η*^2^p = 0.03) and by statistically significantly higher neutrophil (*P* < 0.001, *η*^2^p = 0.02), monocyte (*P* < 0.001, *η*^2^p = 0.02) and lymphocyte (*P* = 0.002, *η*^2^p = 0.01) counts compared to those with a negative toxicology (Fig. 1A, B, C, D, Table S2). Patients testing positive in urine to cannabinoids were also characterized by a statistically significantly lower PLR (*P* = 0.02, *η*^2^p = 0.01) compared to those with a negative toxicology (Fig. 2D–Table S2). A moderator effect for age and for the type of primary psychiatric diagnosis was suggested for the differences in neutrophil counts (*P* = 0.02, *η*^2^p = 0.01 and *P* = 0.02, *η*^2^p = 0.01, respectively). Age also seemed to moderate the findings for the differences in the PLR (*P* < 0.001, *η*^2^p = 0.02) and in lymphocyte counts (*P* < 0.001, *η*^2^p = 0.004); a moderator effect of BZD consumption was additionally suggested for the differences in lymphocyte counts (*P* = 0.05, *η*^2^p = 0.004).

In relation to patients under opioids use, a statistically significantly lower MLR and a statistically significantly higher lymphocyte counts were found when compared to patients with a negative toxicology (*P* = 0.03, *η*^2^p = 0.005 and *P* < 0.001, *η*^2^p = 0.01, respectively) (Fig. 1, Fig. 2C, Table S2). Age seemed to moderate the findings for the differences in the MLR and in lymphocyte counts (*P* < 0.01, *η*^2^p = 0.02 and *P* < 0.01, *η*^2^p = 0.03, respectively); a moderator effect of BZD consumption was also suggested for the differences in lymphocyte counts (*P* = 0.04, *η*^2^p = 0.004).

In addition, patients with a positive toxicology to cocaine were characterized by statistically significantly higher eosinophil counts compared to those testing negative (*P* = 0.004, *η*^2^p = 0.01) (Fig. 1E–Table S2). Again, age seemed to moderate the findings for the differences in eosinophil counts (*P* = 0.01, *η*^2^p = 0.01).

Statistically significant differences were only found for the NLR and for neutrophil and eosinophil counts when comparing patients with a positive toxicology to cannabinoids, opioids or cocaine among themselves. Specifically, patients testing positive to cannabinoids showed the highest NLR and neutrophil counts, and those testing positive to cocaine showed the highest eosinophil counts (Fig. 1, Fig. 2, Table S2).

### 3.4 Blood levels of immune/inflammatory parameters in individuals testing negative vs. those testing positive to cannabinoids, stratified by primary psychiatric diagnosis

A moderator effect of the type of primary psychiatric diagnosis was suggested for the differences in neutrophil counts between patients with a positive toxicology to cannabinoids and those with a negative urine test (see 3.3). This may indicate that neutrophil counts may vary depending on the primary psychiatric diagnosis. Accordingly, we investigated the existence of differences in the levels of neutrophil counts between patients with a negative toxicology to cannabinoids and those with a positive toxicology, after stratifying them according to their primary psychiatric diagnosis (Table 3). Since a statistically significant correlation was found between neutrophil counts and the WBC (*rs* = 0.88, *P* < 0.001), monocyte counts (*rs* = 0.55, *P* < 0.001), basophil counts (*rs* = 0.22, *P* < 0.001) and platelet counts (*rs* = 0.24, P < 0.001), as well as between neutrophil counts and CRP levels (*rs* = 0.29, *P* < 0.001), the NLR (*rs* = 0.74, *P* < 0.001), MLR (*rs* = 0.44, *P* < 0.001) and PLR (*rs* = 0.13, *P* < 0.001), the existence of differences in these parameters were also investigated. Due to a low sample size (see Table 1), patients with a depressive disorder and an adjustment disorder were grouped into one diagnostic category, and patients with a personality disorder and a those with a conduct disorder, into another one. Analyses on patients with a neurodevelopmental disorder, an eating disorder, and an obsessive-compulsive disorder could unfortunately not be performed.

**Table 3:** Blood levels of immune/inflammatory parameters in individuals with a negative urine test vs. those with positive urine test (stratified by primary psychiatric diagnosis).

	Psychotic Disorder (1)		Bipolar Disorder (2)		Depressive + Adjustment Disorder (3)		Personality + Conduct Disorder (4)		SUD (5)	
	Negative n = 312	Positive n = 73	Negative n = 130	Positive n = 23	Negative n = 193	Positive n = 14	Negative n = 53	Positive n = 27	Negative n = 17	Positive n = 29
Blood parameter	Mean (SD)	Mean (SD)	Mean (SD)	Mean (SD)	Mean (SD)	Mean (SD)	Mean (SD)	Mean (SD)	Mean (SD)	Mean (SD)

WBC count (10x^9^/L)	7.16 (2.14)	**8.38 (2.83)∗∗∗**	7.23 (2.51)	8.35 (2.11)	6.95 (1.94)	7.61 (2.17)	7.47 (2.40)	7.88 (2.41)	7.68 (1.39)	7.45 (2.07)
Basophil count (10x^9^/L)	0.04 (0.02)	0.04 (0.02)	0.04 (0.02)	0.04 (0.02)	0.04 (0.02)	0.04 (0.03)	0.04 (0.02)	0.05 (0.02)	0.05 (0.03)	0.04 (0.02)
Neutrophil count (10x^9^/L)	4.22 (1.83)	**5.08 (2.59)∗∗∗**	4.29 (2.16)	4.82 (1.75)	4.04 (1.61)	4.43 (1.59)	4.34 (1.77)	4.23 (1.93)	3.94 (1.29)	4.17 (1.69)
Monocyte count (10x^9^/L)	0.61 (0.22)	**0.71 (0.28)∗∗**	0.59 (0.22)	0.66 (0.21)	0.58 (0.17)	0.59 (0.19)	0.64 (0.25)	0.72 (0.31)	0.63 (0.18)	0.61 (0.15)
Platelet count (10x^9^/L)	236.48 (63.26)	241.18 (47.39)	240.30 (59.48)	247.08 (41.25)	241.82 (59.72)	238.14 (49.33)	235.67 (61.45)	239.18 (59.24)	149.23 (55.62)	236.38 (55.62)
CRP (mg/L)	5.40 (11.26)	7.45 (19.84)	5.67 (9.53)	8.30 (22.73)	6.74 (15.42)	2.31 (1.55)	12.10 (37.08)	5.40 (7.81)	9.26 (12.62)	7.04 (16.59)

Inflammatory ratios	Mean (SD)	Mean (SD)	Mean (SD)	Mean (SD)	Mean (SD)	Mean (SD)	Mean (SD)	Mean (SD)	Mean (SD)	Mean (SD)

NLR	2.30 (1.47)	2.28 (1.55)	2.27 (1.38)	2.06 (1.38)	2.14 (1.35)	1.94 (0.59)	2.19 (1.08)	1.72 (0.95)	1.64 (0.94)	1.99 (1.11)
MLR	0.32 (0.16)	0.31 (0.15)	0.30 (0.27)	0.27 (0.15)	0.29 (0.13)	0.28 (0.13)	0.32 (0.14)	0.28 (0.11)	0.26 (0.12)	0.28 (0.14)
PLR	126.51 (56.72)	107.90 (34.46)	124.49 (44.45)	**97.36 (27.98)∗**	124.66 (57.39)	112.85 (56.46)	122.44 (62.50)	101.55 (42.35)	100.24 (27.18)	109.42 (43.16)

*Abbreviations*: CRP: C-reactive protein; MLR: monocyte-to-lymphocyte ratio; NLR: neutrophil-to-lymphocyte ratio; PLR: platelet-to-lymphocyte ratio; WBC: white blood cell count. Analyses were based on an analysis of covariance (ANCOVA) model with the corresponding blood parameter as the dependent variable, group (negative/positive), as fixed effect variable, and age, consumption of BZD (yes/no), and agent of use (cannabinoids/opioids/cocaine), as covariates. Significant *P*-values are highlighted in bold and marked with an asterisk (i.e., ∗∗∗*P* ≤ 0.001, ∗∗*P* ≤ 0.01, ∗*P* ≤ 0.05).

In the subgroup of patients diagnosed with a psychotic disorder, patients testing positive in urine to cannabinoids showed a statistically significantly higher WBC (*P* < 0.001, *η*^2^p = 0.04) and statistically significantly higher neutrophil (*P* < 0.001, *η*^2^p = 0.03) and monocyte (*P* = 0.002, *η*^2^p = 0.02) counts compared to those testing negative (Table 3).

Patients with a positive toxicology to cannabinoids and a diagnosis of either bipolar disorder, depressive/adjustment disorder, personality/conduct disorder or SUD did not statistically significantly differ in relation to any of the immune/inflammatory parameters assessed when compared with those with a negative toxicology except for the PLR, with patients with a bipolar disorder diagnosis and a positive urine test showing a statistically significantly PLR compared to those testing negative (Table 3).

Statistically significant differences in relation to the WBC, neutrophil, monocyte and platelet counts were not found when comparing patients with a psychotic disorder, bipolar disorder, depressive/adjustment disorder, personality/conduct disorder or SUD among themselves (data not shown).

## 4 Discussion

To the best of our knowledge, this is the first study to date aimed at investigating differences in a set of immune/inflammatory parameters between psychiatric inpatients with a positive urine test to cannabinoids, opioids or cocaine, and those with a negative urine test.

Overall, patients with a positive toxicology were characterized by a significantly higher WBC counts, as well as significantly higher blood levels of neutrophils, monocytes, lymphocytes and eosinophils compared to those with a negative one. The increase in eosinophil counts was particularly evident in the subgroup of patients who tested positive for cocaine. In contrast, patients who tested positive for cannabinoids were characterized by significantly higher WBC, neutrophil and monocyte counts compared to those with a negative toxicology. In addition, a statistically significantly lower MLR was found in the subgroup of patients testing positive in urine to opioids when compared to those with a negative toxicology. These findings are supported by previous existing human studies where an association between cocaine consumption and an increase in eosinophil counts (Berman et al., 2016; Reyes et al., 2018; Gill et al., 2023), opioids consumption and a lower MLR (Banerjee and Sarkar, 1994; Orum et al., 2018), and cannabis use and higher neutrophil counts (Lorenz et al., 2017; Alshaarawy, 2019; Romeo et al., 2023) has been reported, suggesting that cannabinoids, opioids and cocaine may differently impact the immune/inflammatory response system in individuals with and without psychiatric conditions.

Something which makes our study novel is the fact that analyses were not restricted to patients diagnosed with a specific psychiatric disorder, i.e., patients with different psychiatric conditions were included. Thereby, we were able to investigate the influence of the type of primary psychiatric diagnosis on the differences found between patients with a positive toxicology to any of the agents of abuse assessed, and those with a negative toxicology. Interestingly, a moderator effect for the type of psychiatric diagnosis was found for the differences in neutrophil counts in individuals under cannabinoids use, suggesting a differential effect of cannabinoids on neutrophil counts, depending on the primary psychiatric diagnosis. More specific, we found that only among patients with a psychotic disorder diagnosis did cannabinoid users have statistically significantly higher neutrophil counts compared to non-users. Psychotic patients under cannabinoids use were also the only ones characterized by a statistically significantly higher WBC and monocyte counts when compared to non-users.

While the association between cannabis use and non-psychotic psychiatric conditions is still under debate, exogenous cannabis is considered as one of the major environmental risk factors for psychosis. Consumption of cannabis has been repeatedly associated with not only a worsening in psychotic symptoms and/or with an increased risk of psychotic relapse (Kuepper et al., 2011; Haney and Evins, 2016; Bioque et al., 2022) but also, with an increased risk of developing a psychotic disorder in the future (Moore et al., 2007; Di Forti et al., 2019).

The mechanisms for such associations are not fully understood. However, accumulating recent evidence suggests that cannabinoids may increase the risk of psychosis and/or worsen psychotic symptoms by impacting the immune/inflammatory response system of predisposed individuals (Gibson et al., 2020).

Individuals with a psychotic disorder and/or predisposed to are characterized by a dysregulated endocannabinoid system (ECS) (Bioque et al., 2013). The ECS constitutes a complex regulatory network of neurotransmitters, receptors and enzymes that plays a crucial role in maintain inflammatory balance in the human body. While the ECS is widely expressed throughout the body and can be found in almost all organs, the human nervous system and the immune system have interestingly been found to represent the highest expression levels of cannabinoid receptors (Lu and Mackie, 2021). In individuals with psychosis, a decreased expression of the cannabinoid receptor 2 (CB2) and both endocannabinoids synthesizing enzymes (*N*-acyl phosphatidylethanolamine phospholipase and diacylglycerol lipase) have been found when compared to healthy controls (Bioque et al., 2013).

Activation of CB2 receptors has been associated with the inhibition of microglial activation (Olabiyi et al., 2023) and with the blockade of neutrophil and/or monocyte migration (Gómez et al., 2021), that is, with a systemic deactivation of the inflammatory response system. Therefore, abnormalities in the expression and/or of function of CB2 receptors (which may occur in psychosis) may be associated with both microglial activation and with an activation of the inflammatory response system, something which has been repeatedly described in subjects with psychosis (Bioque et al., 2024; Chen et al., 2024).

In psychotic patients under cannabis use, dysregulation of the ECS may be particularly accentuated (Bioque et al., 2013). Therefore, these patients may exhibit heightened activation of their immune/inflammatory response system, as supported by our findings.

The WBC is nowadays considered as a reliable measure of the overall immune system activity (i.e., a high WBC count, or leukocytosis, suggests the existence of an activated immune response system) (Soehnlein et al., 2017). While acute inflammation is characterized by an increase in neutrophil levels, chronic inflammation is also associated with elevated levels of mononuclear cells, such as monocytes (Soehnlein et al., 2017).

Taken all this together, we hypothesize that (recent) cannabinoids use may be associated with a significantly higher (acute) activation of the inflammatory response system in, particularly, individuals diagnosed with a psychotic disorder.

This may have important potential therapeutic implications. An association between an activated inflammatory response system and treatment-resistance has been reported in patients diagnosed with psychotic disorders (Llorca-Bofí et al., 2021; Bioque et al., 2022; Howes and Onwordi, 2023). Interestingly clozapine (i.e., an antipsychotic agent indicated for the management of treatment-resistant schizophrenia) may exert tits action by, among other mechanisms, modulating neutrophil levels (Jones et al., 2023; Llorca-Bofí et al., 2024). Indeed, one of the most serious adverse effects of clozapine is neutropenia, a potentially serious and mortal side effect characterized by a significant reduction in neutrophil counts, which are crucial for, for example, combating infections (Silva et al., 2020). Therefore, we hypothesize modulation of neutrophil counts as a novel treatment strategy in individuals with psychosis under cannabinoids use. Interestingly, accumulating research has suggested clozapine as the first-line treatment for the management of patients with schizophrenia and a history of cannabis use (Brzozowska et al., 2017; Tang et al., 2017).

Although encouraging, our findings must be considered in light of several important limitations. First, immune and/or inflammatory parameters were assessed in the blood, and findings may thus differ from those in the brain. Data about a healthy control group and/or about potential confounding factors, such as body mass index (Pangrazzi et al., 2020), childhood maltreatment and/or the use of psychotropic medication were also not available and therefore, not included in our analyses. However, we controlled for other potential confounding factors, such as type of primary psychiatric diagnosis, illness chronicity, BZD use and/or the presence of comorbid somatic conditions and have made an effort to review the existing literature on the topic, finding a concordance between previous findings, and ours.

Second, information about consumption periodicity was not collected. Thereby, and since measures in urine inform about recent use, findings may vary in patients with a history of chronic use. Unfortunately, immune/inflammatory parameters were assessed at a single timepoint, preventing us from assessing the effects of drug abstinence on these markers over time. In line with this, in a recent study performed by Romeo et al. (2023), tetrahydrocannabinol (THC) cessation was interestingly associated with an increase in inflammatory markers, including WBC count, as well as lymphocyte and monocyte levels, all this correlating with symptomatology of patients with psychosis. However, and as a strength of our study, information about drug consumption relied on quantitative (objective) information, rather than solely on self-reported substance patterns, and a standardized blood sampling procedure in all included patients was implemented, reducing the potential for technical and biological biases (Raouf et al., 2018). Third, data about cannabinoids composition were not collected. *Cannabis sativa* contains not only delta-9-tetrahydrocannabinol (Δ^9^-THC) and/or cannabidiol (CBD), but more than 70 different cannabinoids that are also found in the cannabis products available on the market (Elsohly and Slade, 2005). This may be of relevance, since different animal (Malfait et al., 2000) and human studies have repeatedly suggested that Δ^9^-THC and CBD may exert opposing immunomodulatory effects (Bidwell et al., 2020). Exogenous cannabinoids containing a high percentage of CBD may exert an immunosuppressor and/or anti-inflammatory action, decreasing the numbers of different leukocyte subpopulations, such as monocytes (McDougle et al., 2017; Henshaw et al., 2021).

Both the type and/or concentrations of cannabinoids may differ greatly by, among other factors, the place of origin (Potter et al., 2008; Mehmedic et al., 2010). Specifically in Spain, THC content in the cannabis usually consumed on the streets is between 15 and 28% on average. On the contrary, the mean CBD content has been set at 5% on average (Santos-Álvarez et al., 2021). Accordingly, we assumed that our patients were under consumption of exogenous cannabinoids containing a high percentage of Δ^9^-THC, but unfortunately, we cannot ensure this, something which could have biased our findings and may be responsible of the inconsistencies found in relation to previous existing literature.

Also, the possibility of a type II error (i.e., false negative) should be taken into account when interpreting our findings. Despite including a relatively large total sample size (n = 927), sensitivity subgroup analyses based on the agent of use or on the primary psychiatric diagnosis led to small sample sizes. This could contribute to failing to detect statistically significant differences when actually present. Finally, the retrospective nature of our study, coupled with the relatively small sample size, limited the inferences that could be drawn about causation.

## 5 Conclusions

Overall, our findings suggest that recent cannabinoids use may be associated with a significantly higher activation of the immune/inflammatory response system, particularly in individuals diagnosed with a psychotic disorder. On the contrary, recent cocaine and opioid use may be respectively associated with eosinophilia and with a significantly lower MLR, regardless of the primary psychiatric diagnosis.

We believe our findings may provide important novel valuable insights into how cannabinoids, opioids and cocaine may impact the inflammatory response system of patients diagnosed with different psychiatric conditions and may thus have important clinical and therapeutic implications. For example, modulation of neutrophil counts may represent a novel treatment strategy in individuals with a psychotic disorder and a comorbid cannabis use. However, larger prospective studies, including data about symptoms severity, consumption periodicity and/or about cannabinoids composition are warranted to confirm our findings.

## CRediT authorship contribution statement

**Vicent Llorca-Bofí:** Writing – original draft, Project administration, Methodology, Formal analysis, Data curation, Conceptualization. **Maria Mur:** Writing – review & editing, Supervision, Funding acquisition. **Maria Font:** Writing – review & editing, Project administration, Methodology, Investigation. **Roberto Palacios-Garrán:** Writing – review & editing. **Maite Sellart:** Writing – review & editing. **Enrique del Agua-Martínez:** Writing – review & editing. **Miquel Bioque:** Writing – review & editing, Supervision. **Gara Arteaga-Henríquez:** Writing – original draft, Methodology, Formal analysis, Data curation, Conceptualization.

## Submission declaration

The present work has not been published previously, has not been submitted to another journal while under consideration for Brain, Behavior, and Immunity-Health, and will not be published elsewhere upon acceptance. The manuscript in its current form has been approved by all co-authors.

## Funding

GA-H was supported by the 10.13039/501100000780European Commission, and funded by the European's Union 10.13039/100010661Horizon 2020 research and innovation program under grant agreement numbers 728018 and 754740.

The funding source had no role in the study design, the data collection, the analysis and interpretation of data, the manuscript writing, and in the decision to submit the article for publication. This research did not receive any specific grant from funding agencies in the public, commercial, or not-for-profit sectors.

## Declaration of competing interest

VL-B reported receiving financial support for continuing medical education from 10.13039/501100004231Adamed, 10.13039/501100024679Advanz Pharma, 10.13039/501100006546Angelini, 10.13039/501100012408Casen Recordati, Exeltis, Janssen, 10.13039/501100013327Lundbeck, 10.13039/100014593Neurocrine Biosciences and Rovi outside the submitted work. GA-H reported receiving personal fees from Janssen outside the submitted work.

The authors declare that they have no known competing financial interests or personal relationships that could have appeared to influence the work reported in this paper.

## Data Availability

Data will be made available on request.
