# Supplementary material for: Differences in total and differential white blood cell counts and in inflammatory parameters between psychiatric inpatients with and without recent consumption of cannabinoids, opioids, or cocaine: A retrospective single-center study
Source: Brain Behav Immun Health. 2024 Nov 6;42:100898. doi: 10.1016/j.bbih.2024.100898 (PMC11615885; doi:10.1016/j.bbih.2024.100898)
Supplement: Multimedia component 1 [file mmc1.docx]

**SUPPLEMENTARY INFORMATION**

**Differences in total and differential WBC counts and in inflammatory parameters between psychiatric inpatients with and without recent consumption of cannabinoids, opioids, or cocaine: a retrospective single-center study**

Vicent Llorca-Bofí, Maria Mur, Maria Font, Roberto Palacios-Garrán, Maite Sellart,

Enrique del Agua-Martínez, Miquel Bioque, Gara Arteaga-Henríquez

**Supplementary Table 1.**

| Diagnosis | ICD-10-CM codes | Names |
| --- | --- | --- |
| Psychotic Disorders | F20 | Schizophrenia |
|  | F22 | Persistent delusional disorders |
|  | F23 | Acute and transient psychotic disorders |
|  | F25 | Schizoaffective disorders |
| Depressive Disorders | F32 | Major depressive disorder, single episode |
|  | F33 | Recurrent depressive disorder |
|  | F34.1 | Dysthymic disorder |
| Bipolar Disorder | F30 | Manic episode |
|  | F31 | Bipolar affective disorder |
|  | F34 | Cyclothymia |
| Adjustment disorders | F41 | Other anxiety disorders |
|  | F43 | Reaction to severe stress, and adjustment disorders |
| Personality Disorders (PD) | F60 | Specific personality disorders |
| Non-Alcohol Substance Use Disorder (SUD) | F11 | Mental and behavioural disorders due to use of opioids |
|  | F12 | Mental and behavioural disorders due to use of cannabinoids |
|  | F13 | Mental and behavioural disorders due to use of sedatives or hypnotics |
|  | F14 | Mental and behavioural disorders due to use of cocaine |
| Neurodevelopmental disorders (NDV) | F84 | Autism Spectrum Disorders |
|  | F84.9 | Pervasive Developmental Disorders |
|  | F90 | Attention Deficit and Hyperactivity Disorder |
| Obsessive-compulsive disorder (OCD) | F42 | Obsessive-compulsive disorder |
| Eating disorders | F50 | Eating disorders |
| Conduct disorders | F91 | Conduct disorders |

**Supplementary Table 2.** Blood levels of immune/inflammatory parameters in individuals with a negative urine test vs. individuals with positive urine test (stratified by agent of consumption; cannabinoids, opioids, or cocaine).

|  | Negative  n=714 | Cannabinoids  n=167 | Opioids  n=13 | Cocaine  n=27 | Cannabinoids vs. Opioids vs. Cocaine | |
| --- | --- | --- | --- | --- | --- | --- |
| Blood parameter | Mean (SD) | Mean (SD) | Mean (SD) | Mean (SD) | *P*-value | *η*^2^p |
| WBC count (10x^9^/L) | 7.10 (2.17) | **8.06 (2.50)**^***^ | 7.96 (2.09) | 7.04 (1.90) | 0.09 | 0.02 |
| Basophil count (10x^9^/L) | 0.04 (0.02) | 0.05 (0.02) | 0.05 (0.03) | 0.04 (0.02) | 0.79 | 0.002 |
| Eosinophil count (10x^9^/L) | 0.17 (0.15) | 0.19 (0.13) | 0.26 (0.17) | **0.27 (0.17)^**^** | **0.04** | 0.03 |
| Neutrophil count (10x^9^/L) | 4.16 (1.83) | **4.68 (2.18)**^***^ | 4.17 (1.51) | 3.64 (1.33) | **0.02** | 0.04 |
| Monocyte count (10x^9^/L) | 0.60 (0.21) | **0.68 (0.26)**^***^ | 0.56 (0.14) | 0.63 (0.21) | 0.19 | 0.02 |
| Lymphocyte count (10x^9^/L) | 2.12 (0.73) | **2.46 (0.80)**^**^ | **2.93 (1.17)^***^** | 2.47 (0.76) | 0.13 | 0.02 |
| Platelet count (10x^9^/L) | 237.62 (61.54) | 241.56 (50.82) | 257.08 (49.73) | 243.63 (53.58) | 0.82 | 0.002 |
| CRP (mg/L) | 6.24 (15.47) | 6.97 (17.93) | 4.13 (9.39) | 6.23 (9.25) | 0.83 | 0.002 |
| Inflammatory ratio | Mean (SD) | Mean (SD) | Mean (SD) | Mean (SD) | *P*-value | *η*^2^p |
| NLR | 2.23 (1.38) | 2.12 (1.35) | 1.63 (0.78) | 1.60 (0.76) | **0.03** | 0.03 |
| MLR | 0.31 (0.14) | 0.30 (0.15) | **0.21 (0.07)^*^** | 0.27 (0.11) | 0.07 | 0.03 |
| PLR | 124.72 (54.65) | **106.92 (37.14)^**^** | 106.38 (44.19) | 112.50 (40.99) | 0.87 | 0.001 |

*Abbreviations*: CRP: C-reactive protein; MLR: monocyte-to-lymphocyte ratio; NLR: neutrophil-to-lymphocyte ratio; PLR: platelet-to-lymphocyte ratio; WBC: white blood cell count. Analyses were based on an analysis of covariance (ANCOVA) model with the corresponding blood parameter as the dependent variable, group (negative/positive) as fixed effect variable, and age, consumption of BZD (yes/no), and the type of primary psychiatric diagnosis, as covariates. Significant *P*-values are highlighted in bold and marked with an asterisk (i.e., ^***^*P*≤ 0.001, ^**^*P*≤ 0.01, ^*^*P*≤ 0.05).
